# A pregnancy hormone-cell death link promotes enhanced lupus-specific immunological effects

**DOI:** 10.3389/fimmu.2022.1051779

**Published:** 2022-11-24

**Authors:** Ruchi Sachdeva, Rahul Pal

**Affiliations:** Immunoendocrinology Lab, National Institute of Immunology, Aruna Asaf Ali Marg, Jawaharlal Nehru University (JNU) Complex, New Delhi, India

**Keywords:** pregnancy, human chorionic gonadotropin, autoantibodies, systemic lupus erythematosus, autoimmunity, apoptotic bodies

## Abstract

Women of reproductive age demonstrate an increased incidence of systemic lupus erythematosus, and reproductive hormones have been implicated in disease progression. Additionally, pregnancy can be associated with disease “flares”, the reasons for which remain obscure. While apoptotic bodies are believed to provide an autoantigenic trigger in lupus, whether autoantigenic constituents vary with varying cellular insults, and whether such variations can be immunologically consequential in the context of pregnancy, remains unknown. As assessed by antigenicity and mass spectrometry, apoptotic bodies elicited by different drugs demonstrated the differential presence of lupus-associated autoantigens, and varied in the ability to elicit lupus-associated cytokines from lupus splenocytes and alter the phenotype of lupus B cells. Immunization of tamoxifen-induced apoptotic bodies in lupus-prone mice generated higher humoral autoreactive responses than did immunization with cisplatin-induced apoptotic bodies, and both apoptotic bodies were poorly immunogenic in healthy mice. Incubation of lupus splenocytes (but not healthy splenocytes) with the pregnancy hormone human chorionic gonadotropin (hCG) along with tamoxifen-induced apoptotic bodies (but not cisplatin-induced apoptotic bodies) induced increases in the secretion of lupus-associated cytokines and in the up-modulation of B cell phenotypic markers. In addition, levels of secreted autoantibodies (including of specificities linked to lupus pathogenesis) were enhanced. These events were associated with the heightened phosphorylation of several signaling intermediates. Observations suggest that hCG is a potential disease-promoting co-stimulant in a lupus-milieu; when combined with specific apoptotic bodies, it enhances the intensity of multiple lupus-associated events. These findings deepen mechanistic insight into the hormone’s links with autoreactive responses in lupus-prone mice and humans.

## Introduction

Systemic lupus erythematosus (SLE, or lupus) is a prototypic systemic autoimmune disease characterized by the presence of autoantibodies against almost two hundred self-moieties (1), several of which are associated with pathology (2). Women exhibit a higher incidence of disease (3). Reproductive hormones have been implicated in disease onset and progression (4).

Interestingly, while cell-mediated (Th1/Th17) autoimmune diseases often regress during pregnancy, systemic autoimmune diseases like lupus can often “flare” (5). The administration of human chorionic gonadotropin (hCG, a hormone critical for the sustenance of pregnancy) ameliorates Th1/Th17-mediated autoimmune pathologies in animals (6, 7). Conversely, there exists associative evidence linking hCG with enhanced humoral autoimmune responses in humans. Reports suggest higher levels of hCG in lupus patients (8) and in patients of preeclampsia (9) (a condition with possible autoimmune involvement/etiology), and the injection of hCG in women has been linked with ovarian hyperstimulation syndrome (10), a thrombotic condition in which autoantibodies to phospholipids can arise (11). In a previous study, administration of hCG to lupus-prone mice was shown elicit the generation of autoantibodies (12). Since toll-like receptor (TLR) signaling can contribute to lupus onset and progression (13, 14), the demonstration of the added effects induced by hCG/TLR agonist combinations in that work was of additional relevance.

The current study was prompted by these observations, and also by two additional considerations. The first consideration was that aberrant apoptosis has been implicated in lupus pathogenesis (15, 16), and that apoptotic bodies contain packaged autoantigens, many of which act as endogenous TLR agonists (14, 17). Previous work by this laboratory has described several characteristics of humoral autoreactive responses specifically directed against dying cells in lupus (18–22). The second consideration was that lupus-like symptoms (23, 24) and poly-arthritis (25) have been reported in cancer patients subsequent to the use of certain chemotherapeutic drugs, an indication that there possibly exist differences in the way the products of cell death are perceived by the immune system, depending on the nature of the cytotoxic insult. Whether the constituents of apoptotic bodies can vary, and whether such variations can have a bearing on lupus symptomatology and progression remains inadequately explored. Were this to be true, it could offer alternative explanations of the more general phenomenon of drug-induced lupus (26, 27).

This work first assessed whether apoptotic bodies generated as a result of individual incubation with different apoptosis-inducing drugs differed in their constituents, in the ability to elicit inflammatory responses from splenocytes isolated from healthy mice and lupus-prone mice, as well as in the ability to induce autoreactive responses upon immunization. Whether the co-incubation of splenoctyes with hCG + specific apoptotic bodies results in enhancement in the generation of inflammatory cytokines and autoantibodies, and in the up-modulation of markers on B cells, as well as in heightened proliferative responses, was assessed. Finally, associated changes in cell signaling events were evaluated. The data indicates that, depending on the apoptosis-inducing drug, the constituents of apoptotic bodies vary considerably, and that such variations correlate with apoptotic body-mediated inflammatory and immunological effects both *in vitro* and *in vivo*, more particularly in a lupus milieu. Additionally, hCG provides a “co-stimulatory” effect with particular apoptotic bodies, greatly enhancing signaling as well as the generation of inflammatory cytokines and autoantibodies. These findings, which are of relevance to the phenomena of drug-induced lupus in general and of lupus flares during pregnancy in particular, may also have broader implications, given the overlapping biological functions of hCG and luteinizing hormone (LH).

## Materials and methods

### Ethics

Animal studies were performed in compliance with the U.S. Department of Health and Human Services Guide for the Care and Use of Laboratory Animals. Work was carried out in accordance with the guidelines laid down by the Committee for the Purpose of Control and Supervision of Experiments on Animals of the Government of India. The protocol was approved by the Institutional Animal Ethics Committee of the National Institute of Immunology (IAEC#529/19).

### Mice

Female inbred lupus-prone mice (NZM2410, hereafter referred to as NZM) and healthy mice (FVB/J, hereafter referred to as FVB) were obtained from The Jackson Laboratory. Animals were bred at the Small Animal Facility of the National Institute of Immunology, New Delhi.

### Apoptotic bodies and cell lysate

Lewis lung carcinoma (LLC1) cells were obtained from the American Type Culture Collection (ATCC, Baltimore, U.S.A.). Cultures were maintained in RPMI-1640 supplemented with 10% FCS. All experiments were carried out within 6 months of resuscitation. LLC1 cells were incubated with etoposide (400 μM), curcumin (160 μM), tamoxifen (40 μM), staurosporine (1 μM), cisplatin (400 μM) or 5-fluorouracil (160 μM) (all from Enzo Life Sciences) for 24 hrs to induce apoptosis. Apoptotic bodies were purified as described by Fransen et al., 2009 (28); briefly, cells were centrifuged at 1500 g for 10 mins at 20°C. The supernatant was centrifuged at 15700 g for 50 mins at 20°C. Apoptotic bodies were resuspended in PBS.

4 x 10^6^ LLC1 cells were incubated with 100 µl RIPA buffer containing a protease inhibitor cocktail (20 µl/ml; Calbiochem), phosphatase inhibitor (Thermo Scientific) and 1% Triton X-100 for 20 mins at 4°C. Supernatants, collected after centrifugation at 18000 g for 30 mins at 4°C, were employed as cell lysate.

### Reactivity of reference autoantibodies with moieties in apoptotic bodies

Reactivity of antibodies in reference reagent sera (Center for Disease Control and Prevention, USA) towards moieties in the six preparations of apoptotic bodies was assessed by Western blot, using standard procedures. Briefly, nitrocellulose strips (containing moieties in apoptotic bodies, resolved on SDS-PAGE) were incubated with individual sera, diluted 1:4000 in PBS containing 1% bovine serum albumin (BSA) and 0.1% Tween-20. After further incubation with an appropriately-diluted goat anti-human IgG + IgM-HRP conjugate (Jackson ImmunoResearch), enzyme activity was visualized by enhanced chemiluminescence (ECL; Pierce). Antibodies to β-actin (Santa Cruz Biotechnology) were employed to verify equivalence of protein content.

### Effect of apoptotic bodies on cytokine secretion and B cell surface markers

NZM and FVB splenocytes (0.2x10^6^ cells/well) were individually cultured with the six apoptotic apoptotic body preparations (10 µg/well) or with LLC1 cell lysate (10 µg/ml). The TLR-9 agonist ODN 1826 (20 µM) was employed as positive control. After an incubation of 24 hrs, levels of the lupus-associated cytokines IL-10, TNF-α and IL-6 were estimated in culture supernatants by ELISA (Invitrogen). Cells were dual-stained for CD19 versus other surface markers (MHC II, CD40, CD80, CD83, CD86) using fluorochrome-labeled antibodies (Biolegend). Samples were analyzed on a flow cytometer (BD FACSVerse) and data analyzed using Flow Jo X software (Tree Star, Inc.). Representative gating strategies are depicted in **Supplementary Figure 1**. In some instances, splenocytes were pre-incubated with individual signaling inhibitors (a Stat3 inhibitor (STAT3 Inhibitor XVI, 10μM); a JNK inhibitor (JNK Inhibitor II, 10μM); an ERK inhibitor (PD 98058, 10μM); a p38 inhibitor (SB203580, 10μM)) (Calbiochem) or with the vehicle (DMSO) for 1 hr before incubation with the apoptotic bodies or with ODN 1826.

### Mass spectrometric analysis

Tamoxifen-induced apoptotic bodies and cisplatin-induced apoptotic bodies (25 µg total protein) were individually reduced with 5 mM Tris (2-carboxyethyl) phosphine (TCEP), alkylated with 50 mM iodoacetamide and then digested with trypsin for 16 hrs at 37°C. Digests were “cleaned” using a C18 silica cartridge to remove salt. The dried pellet was resuspended in Buffer A (5% acetonitrile, 0.1% formic acid).

Experiments were performed on an Ultimate 3000 RSLCnano system coupled with an Orbitrap Eclipse. Peptide mixtures (1 μg) were loaded on a C18 Easy-spray column (50 cm, 3.0 μm; ThermoScientific). Peptides were eluted with a 0-40% gradient of Buffer B (80% acetonitrile, 0.1% formic acid) in water at a flow rate of 300 nl/min and injected for MS analysis. LC gradients were run for 100 mins. MS1 spectra were acquired in the Orbitrap (R= 240k; AGQ target = 400 000; Max IT = 50 ms; RF Lens = 30%; mass range = 400-2000; centroid data). Dynamic exclusion was employed for 10 s excluding all charge states for a given precursor. MS2 spectra were collected in the linear ion trap (rate = turbo; AGQ target = 20 000; MaxIT = 50 ms; NCEHCD= 35%).

RAW files (available at https://osf.io/q9vfk/) were analyzed with Proteome Discoverer (v2.2) against the Uniprot mouse reference proteome database. For Sequest and Amanda search, the precursor and fragment mass tolerances were set at 10 ppm and 0.5 Da, respectively. Enzyme specificity was set for trypsin/P (cleavage at the C terminus of “K/R: unless followed by “P”) along with maximum missed cleavages value of two. Carbamidomethyl on cysteine as fixed modification and oxidation of methionine and N-terminal acetylation were considered as variable modifications for the database search. Both peptide spectrum match and protein false discovery rate were set to 0.01 FDR.

### Immunization, ELISA, Western blot

Eight-week-old female NZM and FVB mice were administered three subcutaneous injections of tamoxifen-induced apoptotic bodies or cisplatin-induced apoptotic bodies (20 μg in Incomplete Fruend’s Adjuvant (IFA)), at fortnightly intervals. Control animals received IFA. Sera (at 5 weeks) were assessed for the presence of autoantibodies by ELISA and Western blot. For ELISA, autoantigens (Ro60, RNP A, RNP 68, dsDNA; Arotec Diagnostics Limited) were individually adsorbed onto ELISA plates (1 μg/well) and incubated with pooled sera (diluted 1:1000 in PBS containing 1% BSA and 0.1% Tween-20) for 16 hrs at 4°C. Following further incubation with appropriately diluted goat anti-mouse-HRP antibodies (Jackson ImmunoResearch), enzyme activity was visualized by addition of 3,3′,5,5′-Tetramethylbenzidine (TMB; Invitrogen). For Western blot, nitrocellulose strips (containing moieties in LLC1 lysate, resolved on SDS-PAGE) were incubated with pooled sera, diluted 1:1000 in PBS containing 1% BSA and 0.1% Tween-20. After further incubation with an appropriately-diluted goat anti-mouse HRP conjugate (Jackson ImmunoResearch), enzyme activity was visualized by enhanced chemiluminescence (ECL; Pierce). Antibodies to β-actin (Santa Cruz Biotechnology) were employed to verify equivalence of protein content.

### Effect of hCG-apoptotic body co-incubation on proliferative responses, cytokine secretion, B cell surface markers and autoantibody secretion

2x10^5^ splenocytes isolated from either NZM mice or FVB mice were incubated with hCG (100 IU/ml; Sigma), tamoxifen-induced apoptotic bodies (10 µg/ml), tamoxifen-induced apoptotic bodies + hCG, cisplatin-induced apoptotic bodies (10 µg/ml) or with cisplatin-induced apoptotic bodies + hCG for 24 hrs; concanavalin A (ConA; 1.25 μg/ml), LPS (5 μg/ml) and ODN 1826 (20 µM) were employed as positive controls. ^3^H-thymidine (0.5 μCi/well) was then added, and the extent of cellular incorporation was assessed 18 hrs later. In analogous protocols, levels of the lupus-associated cytokines in supernatants were estimated, and the expression of B cell surface markers assessed, as described above. Supernatants (diluted 1:5 in PBS containing 1% BSA and 0.1% Tween-20) were evaluated for the presence of antibodies by ELISA against standard autoantigens, essentially as described above. Moieties in tamoxifen-induced apoptotic bodies and cisplatin-induced apoptotic bodies were resolved by SDS-PAGE (100 µg protein per gel) and the reactivity of antibodies in the supernatants was assessed by employing Western blot protocols analogous to those described above.

### Effect of hCG-apoptotic body co-incubation on signaling intermediates

4×10^6^ splenocytes isolated from NZM mice or FVB mice were incubated with hCG, tamoxifen-induced apoptotic bodies, tamoxifen-induced apoptotic bodies + hCG, cisplatin-induced apoptotic bodies or with cisplatin-induced apoptotic bodies + hCG. ODN 1826 was employed as positive control. After 30 mins, moieties in cell lysates were resolved by SDS-PAGE (40 µg per lane) and processed for Western blot, using standard protocols. Antibodies (Santa Cruz) reactive towards the following signaling intermediates were employed: Stat3, phospho-Stat3, JNK, phospho-JNK, p38, phospho-p38, ERK1/2, phospho-ERK1/2. After addition of appropriate second antibodies, reactive bands were visualized by enhanced chemiluminescence. Band intensities were quantified employing ImageJ software, and data was plotted as the ratio of phosphorylated/total band intensity.

### Statistical analysis

Data are presented as Mean ± SEM (Standard Error of Mean) unless otherwise stated. The t-test (unpaired) or one-way ANOVA (Holm-Sidak) were used to calculate statistical significance, as appropriate. p values of less than 0.05 were considered significant. Data displayed normal distribution as assessed by the Shapiro-Wilk test.

## Results

### Reactivity of reference autoantibodies with moieties in apoptotic bodies

**Figure 1:**
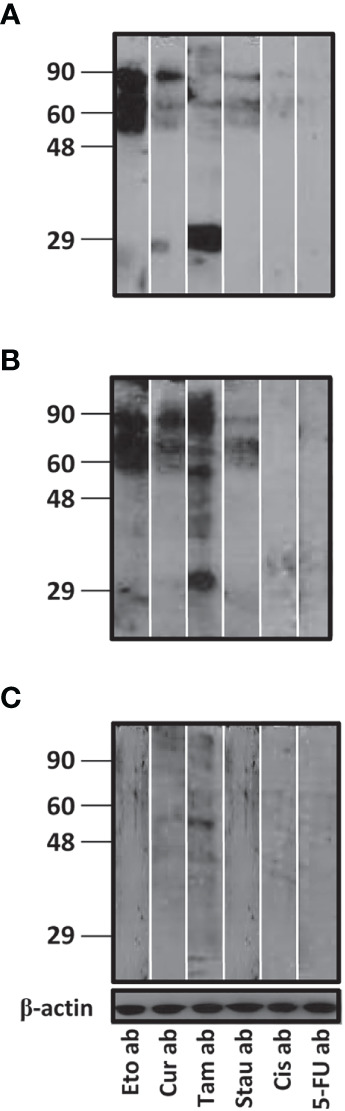
Reactivity of autoantibodies in reference reagent sera with moieties in different apoptotic bodies. Western blots depicting reactivity of autoreactive sera containing antibodies to **(A)** Sm, **(B)** RNPs/La/Sm or **(C)** La towards apoptotic bodies (ab) derived from LLC1 cells upon individual incubation with six cytotoxic drugs: Etoposide (Eto), curcumin (Cur), tamoxifen (Tam), staurosporine (Stau), cisplatin (Cis) and 5-fluorouracil (5-FU). Anti-β-actin antibodies were employed to verify equivalence of loading. Positions of molecular weight markers (Kda) are indicated.

On Western blots, antibodies in reference sera (certified to contain antibodies to the autoantigens Sm, RNPs/La/Sm or La, but which could also contain antibodies to other autoantigens, several of which lie in the 49 Kda – 90 Kda range) displayed relatively enhanced reactivity towards moieties in apoptotic bodies generated upon the action of etoposide, curcumin or tamoxifen. Tamoxifen-induced apoptotic bodies demonstrated reactivity towards antibodies in all three sera to varying degrees, while moieties in apoptotic bodies generated upon the action of 5-fluorouracil and cisplatin were non-reactive towards antibodies in all three sera, and sera containing antibodies against Smith and RNP/La/Smith demonstrated moderate reactivity towards staurosporine-induced apoptotic bodies (**Figure 1**). Autoantigenic moieties present in different apoptotic bodies therefore varied quite considerably.

### Effect of apoptotic bodies on cytokine secretion and B-cell phenotype

**Figure 2:**
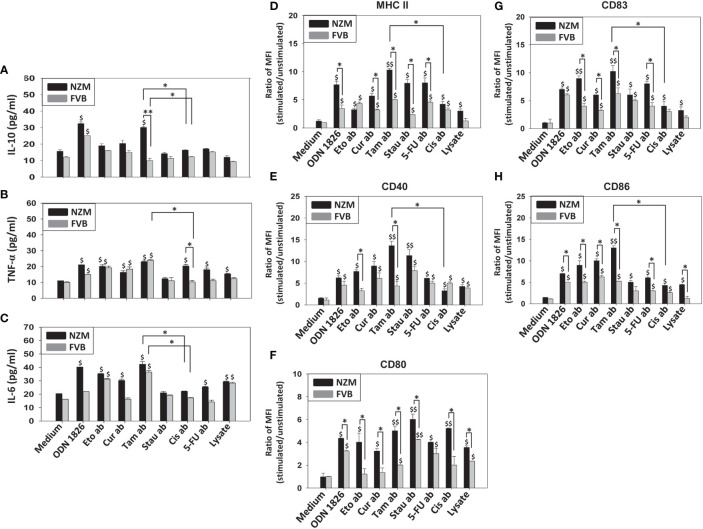
Effect of apoptotic bodies on cytokine secretion and B-cell phenotype. Splenocytes from NZM (n = 3) and FVB (n = 3) mice were individually incubated with the six apoptotic body (ab) preparations induced by etoposide (Eto), curcumin (Cur), tamoxifen (Tam), staurosporine (Stau), cisplatin (Cis) or 5-fluorouracil (5-FU)), or with cell lysate. ODN 1826 was employed as positive control. **(A-C)** IL-10, TNF-α and IL-6 concentrations in culture supernatants. For purposes of convenience and clarity, only comparisons between Tam-induced apoptotic bodies (Tam ab) and Cis-induced apoptotic bodies (Cis ab) have been highlighted. **(D-H)** Flow cytometric analysis of MHC II, CD40, CD80, CD83 and CD86 on CD19^+^ cells. Bars represent arithmetic means ± SEM of ratios (stimulated cells/unstimulated cells) of Mean Fluorescence Intensity (MFI). ^$^p ≤ 0.05, ^$$^p ≤ 0.01 versus Medium; *p ≤ 0.05, **p ≤ 0.01 by ANOVA.

Several apoptotic bodies elicited the secretion of significant levels of IL-10, TNF-α and IL-6 from splenocytes derived from both FVB and NZM mice. Tamoxifen-induced apoptotic bodies elicited the highest cytokine responses. For purposes of convenience and clarity, only comparisons between tamoxifen-induced apoptotic bodies and cisplatin-induced apoptotic bodies have been highlighted (**Figures 2A–C**). Multiple signaling events were differentially responsible for cytokine secretion in NZM splenocytes. Upon incubation with tamoxifen-induced apoptotic bodies, the Stat3 inhibitor XVI decreased the secretion of IL-6, the JNK II inhibitor led to decreased secretion of TNF-α, and addition of SB 203580 (a p38 inhibitor) resulted in diminished secretion of IL-10, TNF-α and IL-6. Upon incubation with cisplatin-induced apoptotic bodies, PD 98058 (an ERK inhibitor) decreased the basal secretion of IL-10, and SB 203580 decreased the secretion of IL-6 as well as IL-10 (**Supplementary Figure 2**).

Interestingly, while all apoptotic bodies enhanced levels of phenotypic markers on B cells derived from both FVB and NZM mice (to varying degrees), for many markers, several apoptotic bodies elicited higher phenotypic changes on B cells derived from NZM mice than from FVB mice. Additionally, on B cells derived from NZM mice, in comparison with cisplatin-induced apoptotic bodies, tamoxifen-induced apoptotic bodies significantly elevated levels of MHC II, CD40, CD83 and CD86 (**Figures 2D–H**). Tamoxifen-apoptotic body induced expression of MHC II and CD86 significantly decreased upon addition of the Stat3 inhibitor XVI, and addition of JNK II inhibitor led to lowered expression of MHC II, CD40, CD80 and CD86, while addition of PD 98058 (an ERK inhibitor) led to decreased expression of CD83 and CD86. SB 203580 (a p38 inhibitor) caused a decrease in expression of MHC II, CD40, CD83 and CD86 (**Supplementary Figure 3**).

While cell lysate also stimulated the release of TNF-α and IL-6 and induced the up-modulation of cell surface markers on B cells (possibly attributable to the presence of many self-antigens/potential autoantigens, many of which behave as endogenous TLR ligands) to an extent, tamoxifen-induced apoptotic bodies were generally additionally inflammatory.

### Mass spectrometric analysis of tamoxifen-induced apoptotic bodies and cisplatin-induced apoptotic bodies

**Figure 3:**
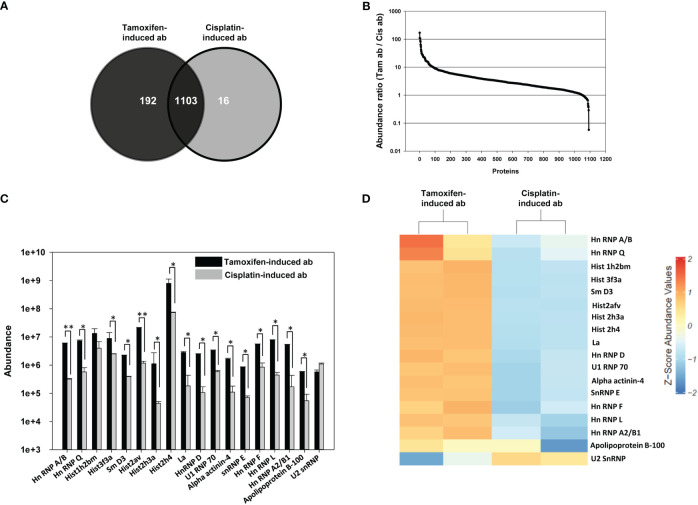
Mass spectrometric analysis of tamoxifen-induced apoptotic bodies and cisplatin-induced apoptotic bodies. Tamoxifen-induced apoptotic bodies (ab) and cisplatin-induced apoptotic bodies (ab) were individually reduced with TCEP, alkylated with 50 mM iodoacetamide and then digested with trypsin. Mass spectrometric analysis was then carried out. **(A)** Venn diagram depicting the average number of unique as well as common proteins in tamoxifen-induced ab (dark gray) and cisplatin-induced ab (light gray) in three different experiments. **(B)** Abundance ratios (Tamoxifen-induced (Tam) ab to cisplatin-induced (Cis) ab) for common proteins. **(C)** The abundance of prominent lupus-related autoantigens in Tam-induced ab and Cis-induced ab. *p ≤ 0.05, **p ≤ 0.01 by the t-test (unpaired). Bars represent arithmetic means ± SEM of three different experiments. **(D)** Heat-map comparison of prominent lupus-related autoantigens in Tam-induced ab and Cis-induced ab.

Tamoxifen-induced apoptotic bodies contained a higher number of proteins than did cisplatin-induced apoptotic bodies, as determined by ESI-LC-MS/MS mass spectrometric analysis. Several proteins were common to both apoptotic body preparations, while others were unique to each preparation (**Figure 3A**). **Figure 3B** depicts abundance ratios (tamoxifen-induced apoptotic bodies to cisplatin-induced apoptotic bodies) for the common proteins. The differential abundance of lupus-associated autoantigens common to tamoxifen-induced apoptotic bodies and cisplatin-induced apoptotic bodies was assessed employing DAVID software and functional annotation bioinformatics microarray analysis. Several prominent autoantigens (including several histones, ribonucleoproteins like U1 RNP 70, Hn RNP D, Hn RNP F, Hn RNP L, SnRNP E, Sm and La, as well as alpha actinin) were present in greater abundance in tamoxifen-induced apoptotic bodies than in cisplatin-induced apoptotic bodies (**Figure 3C**), a fact also made clear by heat-maps constructed upon hierarchical clustering of significant autoantigens after averaging Z-score abundance values (**Figure 3D**).

### Immunogenicity of apoptotic bodies

**Figure 4:**
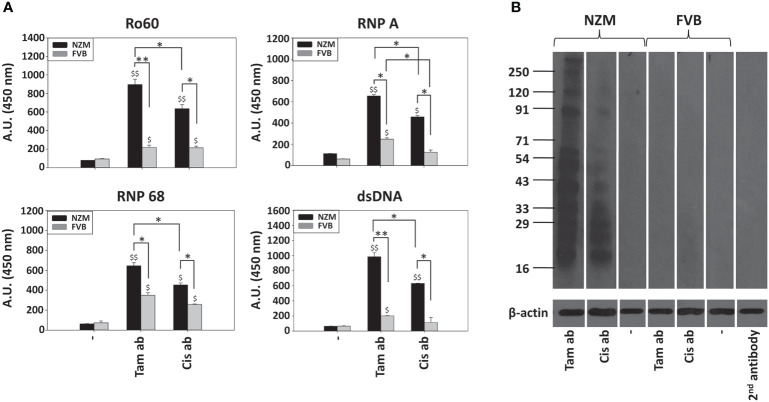
Immunogenicity of tamoxifen-induced apoptotic bodies and cisplatin-induced apoptotic bodies. Eight-week-old NZM and FVB female mice (n = 6) were immunized with either tamoxifen-induced apoptotic bodies (Tam ab) or cisplatin-induced apoptotic bodies (Cis ab). Control mice (“-”) received adjuvant. **(A)** Reactivity of pooled antisera against the autoantigens Ro60, RNP A, RNP 68 and dsDNA by ELISA. Bars represent arithmetic means ± SEM of Antibody Units (A.U.; Dilution Factor x O.D. (450 nm)) of three different experiments. ^$^p ≤ 0.05, ^$$^p ≤ 0.01 versus respective control mice; *p ≤ 0.05, **p ≤ 0.01, by ANOVA. **(B)** Reactivity of antisera against moieties in LLC1 lysate on Western blot. 2^nd^ antibody: Reactivity of the anti-mouse-HRP conjugate, as negative control. Anti-β-actin antibodies were employed to verify equivalence of loading. Positions of molecular weight markers (Kda) are indicated.

While immunization of both NZM mice and FVB mice with tamoxifen-induced apoptotic bodies resulted in the generation of antibodies to Ro60, RNP A, RNP 68 as well as to dsDNA, responses in NZM mice were significantly higher. Immunization of NZM mice with cisplatin-induced apoptotic bodies also resulted higher antibody responses against the autoantigens than did immunization in FVB mice. Additionally, immunization of NZM mice with tamoxifen-induced apoptotic bodies resulted in significantly higher levels of autoantibodies against all autoantigens than did immunization with cisplatin-induced apoptotic bodies (**Figure 4A**).

Tamoxifen-induced apoptotic bodies, when immunized in NZM mice, elicited the generation of autoantibodies to a wide spectrum of moieties in cell lysate. Cisplatin-induced apoptotic bodies did not elicit the generation of autoantibodies of equivalent specificity when immunized in NZM mice. Immunization of FVB mice with either tamoxifen-induced apoptotic bodies or cisplatin-induced apoptotic bodies did not result in discernible autoreactivity (**Figure 4B**). These findings indicate that tamoxifen-induced apoptotic bodies exhibit enhanced immunogenicity in a lupus milieu.

### Effects of hCG-apoptotic body co-stimulation on proliferative responses, cytokine secretion and B cell phenotype

Tamoxifen-induced apoptotic bodies induced higher proliferative responses in splenocytes isolated from NZM mice than from FVB mice, with responses being respectively higher than those elicited by cisplatin-induced apoptotic bodies in both strains. The addition of hCG along with tamoxifen-induced apoptotic bodies as well as with cisplatin-induced apoptotic bodies enhanced levels of proliferation over individual components, an effect restricted to splenocytes derived from NZM mice. Further, tamoxifen-induced apoptotic bodies + hCG induced significantly higher proliferative responses than did cisplatin-induced apoptotic bodies + hCG in both murine strains (**Supplementary Figure 4**).

**Figure 5:**
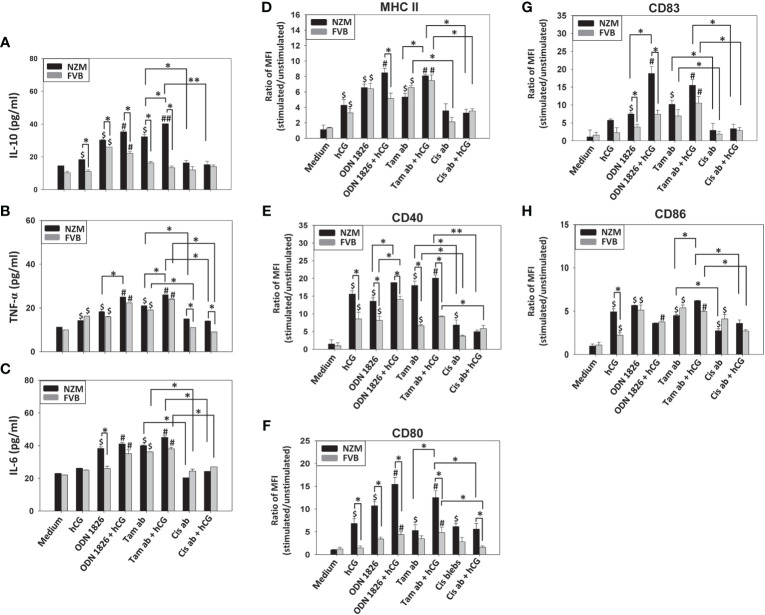
Effects of hCG-apoptotic body co-incubation on cytokine secretion and B cell phenotype. Splenocytes from NZM (n = 3) and FVB (n =3) mice were incubated with hCG, tamoxifen-induced apoptotic bodies (Tam ab), Tam ab + hCG, cisplatin-induced apoptotic bodies (Cis ab) or with Cis ab + hCG. ODN 1826 was employed as positive control. **(A-C)** IL-10, TNF-α and IL-6 concentrations in culture supernatants. **(D-H)** Flow cytometric analysis of MHC II, CD40, CD80, CD83 and CD86 on CD19^+^ cells. Bars represent arithmetic means ± SEM of ratios (stimulated cells/unstimulated cells) of Mean Fluorescence Intensity (MFI). ^$^p ≤ 0.05 versus Medium. ^#^p ≤ 0.05, ^##^p ≤ 0.01 versus hCG; *p ≤ 0.05, **p ≤ 0.01 by ANOVA. For purposes of clarity, significances versus Medium are not shown for conditions containing ODN 1826/apoptotic bodies + hCG.

While hCG induced some secretion of IL-10 and TNF-α from splenocytes, incubation of NZM splenocytes with tamoxifen-induced apoptotic bodies + hCG (but not with cisplatin-induced apoptotic bodies + hCG) resulted in enhanced secretion of these cytokines, compared to when the two moieties were individually incubated. Incubation of FVB splenocytes with either tamoxifen-induced apoptotic bodies + hCG or cisplatin-induced apoptotic bodies + hCG did not result in such additive inflammatory effects. Differences between tamoxifen-induced apoptotic bodies and cisplatin-induced apoptotic bodies were also apparent (both in the absence and presence of hCG, and in both murine strains) when IL-6 was measured (**Figures 5A–C**). Similarly, while hCG induced the up-modulation of some markers on B cells, incubation with tamoxifen-induced apoptotic bodies + hCG enhanced levels of all measured phenotypic markers (MHC II, CD40, CD80, CD83, CD86) on B cells compared with incubation with cisplatin-induced apoptotic bodies + hCG in both strains of mice. Specifically on NZM B cells, incubation with tamoxifen-induced apoptotic bodies + hCG also led to increase in the expression of MHC II and CD80, compared to when the two moieties were individually incubated; further, enhancement in the levels of CD40 and CD80 were significantly greater than on B cells derived from FVB mice. Similar effects with hCG were not observed with cisplatin-induced apoptotic bodies, on either NZM B cells or FVB B cells (**Figures 5D–H**).

### Effects of hCG-apoptotic body co-stimulation on the secretion of autoantibodies

**Figure 6:**
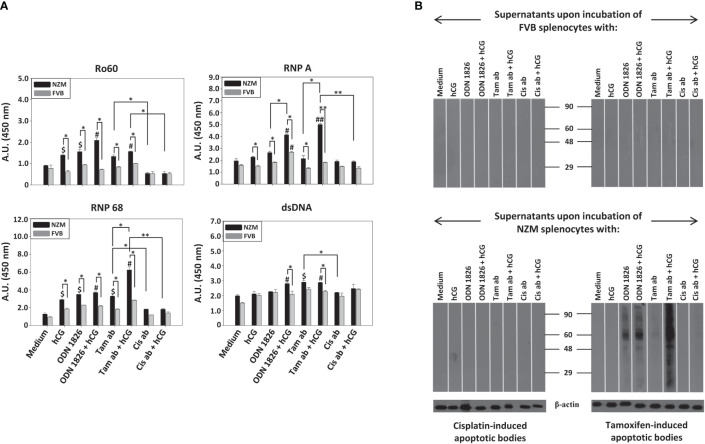
Effect of hCG-apoptotic body co-incubation on autoantibody secretion. Splenocytes from NZM (n = 3) and FVB (n = 3) mice were incubated with hCG, tamoxifen-induced apoptotic bodies (Tam ab), Tam ab + hCG, cisplatin-induced apoptotic bodies (Cis ab) or Cis ab + hCG. ODN 1826 was employed as positive control. **(A)** Reactivity of antibodies in culture supernatants towards Ro60, RNP A, RNP 68 and dsDNA by ELISA. Bars represent arithmetic means ± SEM of Antibody Units (A.U.; Dilution Factor x O.D. (450 nm)). ^$^p ≤ 0.05 versus Medium; ^#^p ≤ 0.05, ^##^p ≤ 0.01 versus hCG; *p ≤ 0.05, **p ≤ 0.01 by ANOVA. For purposes of clarity, significances versus Medium are not shown for conditions containing ODN 1826/apoptotic bodies + hCG. **(B)** Reactivity of antibodies in supernatants towards moieties in cisplatin-induced apoptotic bodies and tamoxifen-induced apoptotic bodies on Western blot. Anti-β-actin antibodies were employed to verify equivalence of loading. Positions of molecular weight markers (Kda) are indicated.

Tamoxifen-induced apoptotic bodies (but not cisplatin-induced apoptotic bodies) elicited the generation of antibodies to RNP 68 and dsDNA when incubated with NZM splenocytes, but not when incubated with FVB splenocytes. Interestingly, hCG also induced the generation of antibodies to Ro60 and RNP 68 when incubated with NZM splenocytes, but not when incubated with FVB splenocytes. The combination of tamoxifen-induced apoptotic bodies and hCG, when incubated with NZM splenocytes (but not with FVB splenocytes) induced increases in the secretion of antibodies to RNP A and RNP 68 over conditions when tamoxifen-induced apoptotic bodies or hCG were individually incubated. Such effects were not observed when hCG and cisplatin-induced apoptotic bodies were co-incubated, on either NZM splenocytes or FVB splenocytes (**Figure 6A**). On Western blot, while tamoxifen-induced apoptotic bodies induced a low level of autoreactivity (apparent only when tamoxifen-induced apoptotic bodies themselves were employed as target) when incubated with NZM splenocytes (but not with FVB splenocytes), hCG was incapable of eliciting autoreactive antibodies on its own. This was in apparent contrast with reactivity the hormone induced against some autoantigens, indicated above. Such discrepancies are not unusual, and could be attributed to epitope destruction during blotting procedures. The combinations of tamoxifen-induced apoptotic bodies and hCG, when added to NZM splenocytes (but not to FVB splenocytes), elicited antibodies that bound a wide spectrum of moieties in tamoxifen-induced apoptotic bodies. Such antibodies were non-reactive towards moieties in cisplatin-induced apoptotic bodies, once again underscoring autoantigenic differences between constituents in the two apoptotic bodies. The combination of cisplatin-induced apoptotic bodies and hCG was incapable of inducing similar autoreactive responses (**Figure 6B**). These results imply that hCG can act as a “co-stimulant” in a lupus milieu, inducing the heightened generation of autoantibodies, but only in conjunction with certain apoptotic bodies.

### Effect of hCG-apoptotic body co-stimulation on signaling intermediates

**Figure 7:**
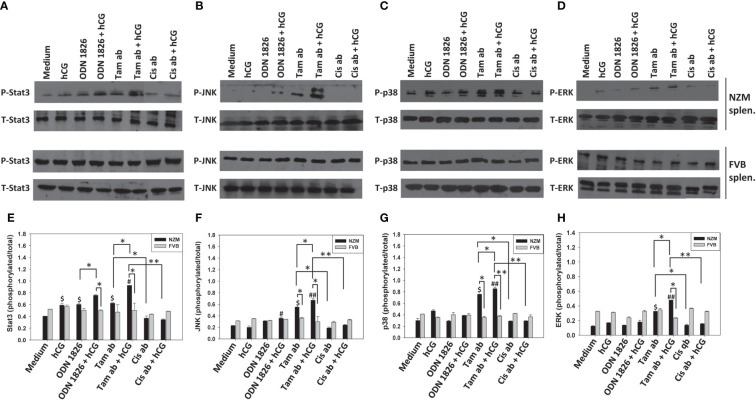
Effect of hCG-apoptotic body co-incubation on signaling intermediates. Splenocytes (splen.) from NZM (n = 3) and FVB (n = 3) mice were incubated with hCG, tamoxifen-induced apoptotic bodies (Tam ab), Tam ab + hCG, cisplatin-induced apoptotic bodies (Cis ab) or Cis ab + hCG. ODN 1826 was employed positive control. **(A–D)** Representative Western blots depicting phosphorylated (P) and total (T) Stat3, JNK, p38 and ERK1/2. **(E–H)** Ratios of respective phosphorylated-to-total signal band intensities by ImageJ analysis; each graph corresponds to the Western blots above it. Bars represent arithmetic means ± SEM. ^$^p ≤ 0.05 versus Medium. ^#^p ≤ 0.05, ^##^p ≤ 0.01 versus hCG; *p ≤ 0.05, **p ≤ 0.01 by ANOVA. For purposes of clarity, significances versus Medium are not shown for conditions containing ODN 1826/apoptotic bodies + hCG.

In NZM splenocytes (but not in FVB splenocytes), tamoxifen-induced apoptotic bodies (but not cisplatin-induced apoptotic bodies) induced phoshorylation of Stat3, JNK, p38 and ERK1/2. Incubation of hCG along with tamoxifen-induced apoptotic bodies on NZM splenocytes (but not on FVB splenocytes) led to increases in the phosphorylation levels of all four signaling intermediates over those observed with tamoxifen-induced apoptotic bodies or hCG alone. Such effects were not observed when cisplatin-induced apoptotic bodies were employed along with hCG, on either NZM or FVB splenocytes (**Figure 7**).

## Discussion

Systemic lupus erythematosus is an autoimmune disease characterized by the presence of antibodies against multiple self-moieties, including those targeting lipids, proteins and nucleic acids (1). Levels of several pro-inflammatory cytokines are elevated (29), and glomerulonephritis is a major pathological outcome (30). Several studies suggest that aberrant apoptosis plays a major role in lupus onset, and that abnormal clearance of apoptotic cells can result their accumulation in germinal centers (15, 16, 31, 32). Since apoptotic bodies contain autoantigens (33, 34), these events are believed to contribute to a break in self-tolerance. Pioneering observations indicated that anti-self humoral immune responses were generated upon the intravenous administration of apoptotic thymocytes in normal mice (35). Subsequent experiments revealed that immunization of either healthy or lupus-prone mice with apoptotic thymocytes *via* other routes (along with either an adjuvant or syngeneic dendritic cells) resulted in divergent immunopathological consequences; while healthy mice did not generate significant anti-self responses, lupus-prone mice developed high levels of autoantibodies and demonstrated evidence of kidney disease. Increasing the number of apoptotic cells during immunization could compensate for the lack of adjuvant (36).

Post-apoptotic events can be influenced by the nature of apoptosis-inducing stimuli. For example, whether cells die as a consequence of etoposide or staurosporine can alter the efficiency of phagocytic uptake (37). Auto-reactive antibodies are frequently observed in patients of cancer (38, 39) a phenomenon at least partially attributable to the use of particular apoptosis-inducing chemotherapeutic agents. For example, rheumatic symptoms are observed in cancer patients receiving cyclophosphamide/5-fluorouracil combination chemotherapy (40), and the administration of tamoxifen in cancer patients can be associated with the onset of lupus-like symptoms and polyarthritis, and the generation of anti-nuclear antibodies and anti-Ro antibodies (23–25). While the administration of tamoxifen in lupus-prone mice has been shown to lead to decreased autoantibody levels and an amelioration of symptoms (41–43), attributed to the anti-estrogenic effects of the drug, studies in SLE patients suggest a possible worsening of disease (44).

In this work, autoantigenic content of apoptotic bodies, as well as their inflammatory potential and the ability to influence B cell phenotype, was shown to be influenced by the nature of the chemotherapeutic drug. Specific cytokines and cell-surface markers were chosen for study for a specific purpose. IL-10, TNF-α and IL-6 have been implicated in lupus pathogenesis (45–47). In addition, several co-stimulatory molecules are known to be up-modulated on B cells as well as on other antigen presenting cells in lupus patients, and many of these changes are associated with the production of pathogenic antibodies (48–52). In this study, apoptotic bodies arising upon the action of several drugs induced significant inflammatory responses and up-modulated several phenotypic markers on B cells, and in many instances, effects were more pronounced on splenoctyes isolated from NZM mice. While the two apoptotic body preparations (tamoxifen-induced apoptotic bodies and cisplatin-induced apoptotic bodies) that appeared most distinct in terms of the three readouts were chosen for further study for logistical reasons, investigation into the properties of other apoptotic body preparations would no doubt yield additional clues, and such studies are on-going.

Autoantigens contained within apoptotic bodies can act as TLR agonists which can trigger MAP kinase signaling (53). Inhibitors for the three MAP kinase signaling pathways were therefore employed, and the effects of a STAT3 inhibitor also assessed, given the convergence/cross-influence of STAT3 and MAP kinase-mediated signaling (54–56). While the p38 inhibitor, the Stat3 inhibitor, as well as the JNK inhibitor all inhibited different cytokines to varying degrees, the p38 inhibitor had the most prominent anti-inflammatory effects, reducing the levels of all three measured cytokines. The signaling inhibitors had variable effects on tamoxifen-induced apoptotic body influenced up-modulation of B cell surface markers as well; the p38 inhibitor and the JNK inhibitor had the broadest inhibitory capabilities. These studies suggest that apoptotic bodies trigger multiple signalling events to drive lupus-relevant outcomes in lupus splenocytes.

Mass spectrometric analysis revealed that abundance scores of several HnRNPs, SnRNPs, as well as of La and Sm, were higher in tamoxifen-induced apoptotic bodies than in cisplatin-induced apoptotic bodies. These moieties constitute prominent lupus autoantigens (57). In addition, scores for several histones and for alpha-actinin, were also higher in tamoxifen-induced apoptotic bodies, moieties against which tolerance is also compromised in lupus (58–61). Correlating with these findings was the fact that tamoxifen-induced apoptotic bodies exhibited an enhanced ability to induce pathogen-associated autoantibodies upon immunization, particularly in lupus-prone mice. These results suggest that quantitative and qualitative apoptotic body attributes influence lupus-relevant readouts both *in vitro* and *in vivo*.

The fact that reproductive hormones can influence the progression and severity of lupus has been known for a while (4, 62–64). Of relevance to the current work, studies also potentially link hCG to lupus (8, 65) and to other conditions with overlapping pathology and/or serology (9, 10, 66). Contrary to its ameliorating effects on T cell-driven autoimmune diseases (6, 7), the administration of hCG in lupus-prone (but not healthy) mice stimulates the production of autoantibodies and adversely affects life-span (12). An enhanced effect of hCG and Toll-like receptor-7 (TLR-7)/TLR-9 agonist combinations on the up-modulation of co-stimulatory markers on B cells, as well as on the secretion of lupus-associated cytokines and autoantibodies, has also been described (12), an observation of significance since TLR signaling has been linked with lupus progression (13, 14, 67). Besides its presence during pregnancy (during which lupus flares can be observed), other observational and experimental data therefore also suggest that the presence of hCG is associated with/can induce a Th2 immunological skew, supportive of autoantibody production.

The current study provides physiological context and mechanistic insight into the potential roles hCG can play in lupus. Recombinant hCG was intentionally not employed in these studies since it can exhibit alterations in the extent and pattern of glycosylation, and carbohydrates are known to affect the biological activity of the hormone. Since the phenomenon being reported upon was as yet undescribed, a physiological, native hormone preparation was employed. While the purity of hCG employed in these studies (13,000 IU/mg) was equivalent to that reported for commercial recombinant hCG preparations, carrying out similar experiments with the latter would no doubt be instructive.

Apoptotic bodies are believed to constitute the original antigenic sin in lupus. In multiple readouts, including activation of several signaling cascades, as well as the production of inflammatory cytokines and autoantibodies, specific enhancements were observed between tamoxifen-induced apoptotic bodies (but not cisplatin-induced apoptotic bodies) and hCG. That such effects were restricted to the lupus-genotype was particularly noteworthy. An appropriate and logical extension of the current work would be to investigate whether different preparations of apoptotic bodies elicit differential autoreactive responses, as well as immunopathology, when administered along with hCG *in vivo*; such experiments are ongoing.

The observation that data displays some variation across experiments in some instances was possibly attributable to a few factors. The studies described here were carried out at different times for logistical and practical reasons. Lupus-onset in NZM 2410 mice is age-dependent, and the age of mice employed could not be controlled to within a couple of weeks. Coupled with this fact, there could have been other unanticipated effects. Though NZM 2410 mice express lupus-like disease at higher penetrance than other lupus-prone strains, the incidence of overt disease reaches about 80% only at 200 days, or at about 28 weeks; incidence never reaches 100%, particularly in females (68). Experiments reported here were carried on 6 to 8-week-old animals which were not pre-screened for overt disease, since even animals destined to develop disease may not show the presence of autoantibodies at that time. The “random” selection of animals at different times may contribute to an extent to the observed variability. It remains to be determined if conducting these experiments on older animals (which have been pre-screened for overt disease) circumvents some of these issues; such studies are on-going.

While greater appreciation of the molecular basis of the effects that hCG-apoptotic body combinations induce could provide leads to a deeper understanding of pregnancy-associated disease “flares”, current observations may have broader implications, since a role for LH can now be envisaged. LH and hCG share a common receptor and mediate similar downstream events (69, 70). An ovulation-inducing LH surge occurs during each menstrual cycle in fertile women. Given that women of reproductive age exhibit high disease incidence (3, 71), were LH to exhibit similar agonistic capabilities, it would serve to further augment theories of lupus onset and progression in non-pregnant SLE patients belonging to this age group.

## Data availability statement

The data presented in the study is deposited in the OSF repository (https://osf.io/) and can be accessed via the following link: https://osf.io/q9vfk/.

## Ethics statement

The animal study was reviewed and approved by the Institutional Animal Ethics Committee of the National Institute of Immunology (IAEC#529/19).

## Author contributions

RS and RP conceived the study and designed the experiments. RS carried out the experiments and drafted the manuscript, RP edited and finalized the manuscript. All authors contributed to the article and approved the submitted version.

## Funding

This work was supported by an extra-mural grant from the Department of Biotechnology, Government of India to RP (Grant number BT/PR/15020/Med/30/587/2010) and by core grants from the National Institute of Immunology, New Delhi, India.

## Acknowledgments

The authors thank Mr. Ashok Kumar and Ms. Shanta Sen for technical assistance.

## Conflict of interest

The authors declare that the research was conducted in the absence of any commercial or financial relationships that could be construed as a potential conflict of interest.

## Publisher’s note

All claims expressed in this article are solely those of the authors and do not necessarily represent those of their affiliated organizations, or those of the publisher, the editors and the reviewers. Any product that may be evaluated in this article, or claim that may be made by its manufacturer, is not guaranteed or endorsed by the publisher.
