# Supplementary material for: A pregnancy hormone-cell death link promotes enhanced lupus-specific immunological effects
Source: Front Immunol. 2022 Nov 24;13:1051779. doi: 10.3389/fimmu.2022.1051779 (PMC9730325; doi:10.3389/fimmu.2022.1051779)
Supplement: Supplementary file 1 [file DataSheet_1.pdf]

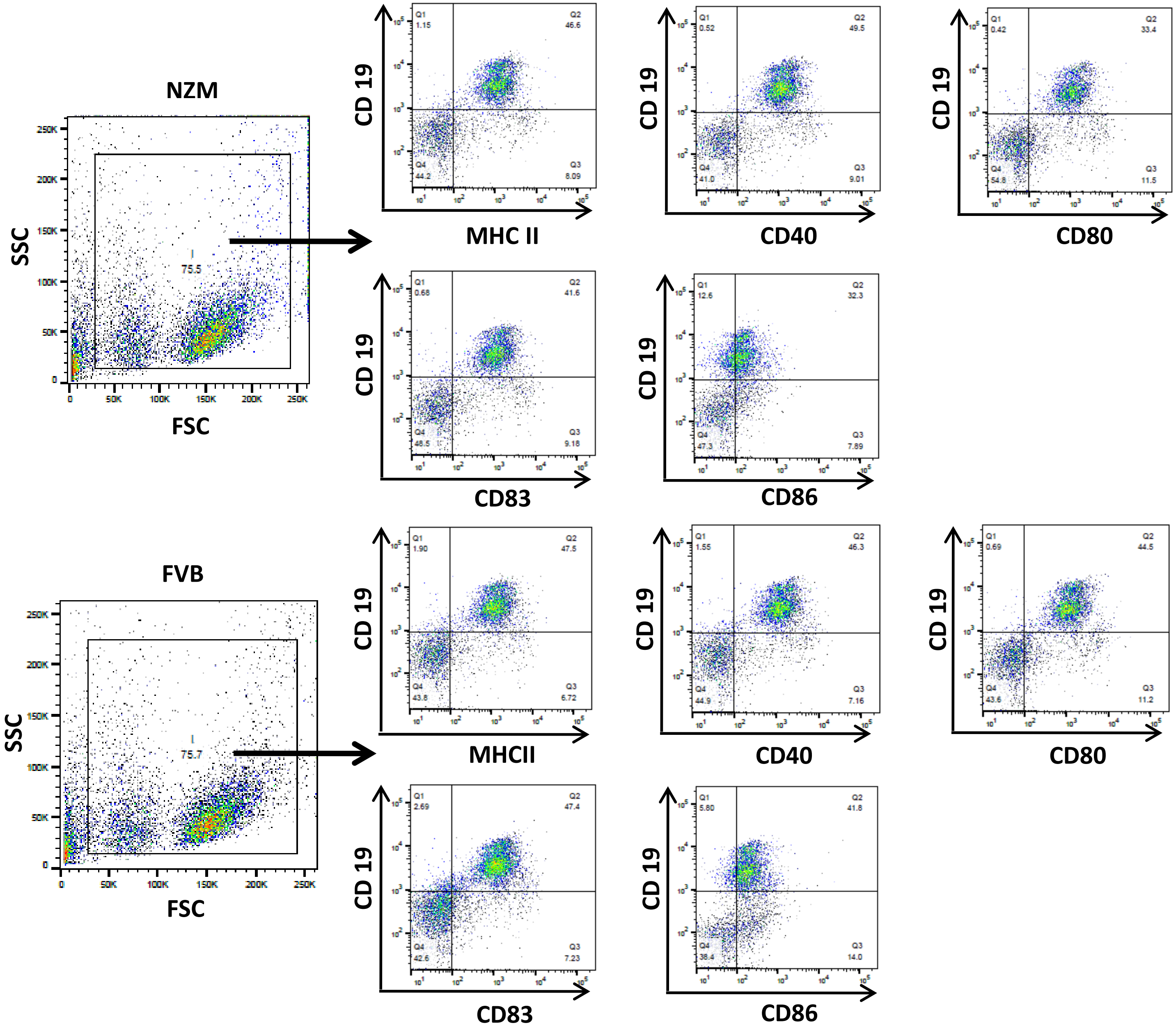

Supplementary Figure 1. Representative flow cytometric analysis. Splenocytes from NZM and FVB mice were dual-stained for CD19 versus other surface markers - MHC II, CD40, CD80, CD83 or CD86; representative gating strategies are depicted.

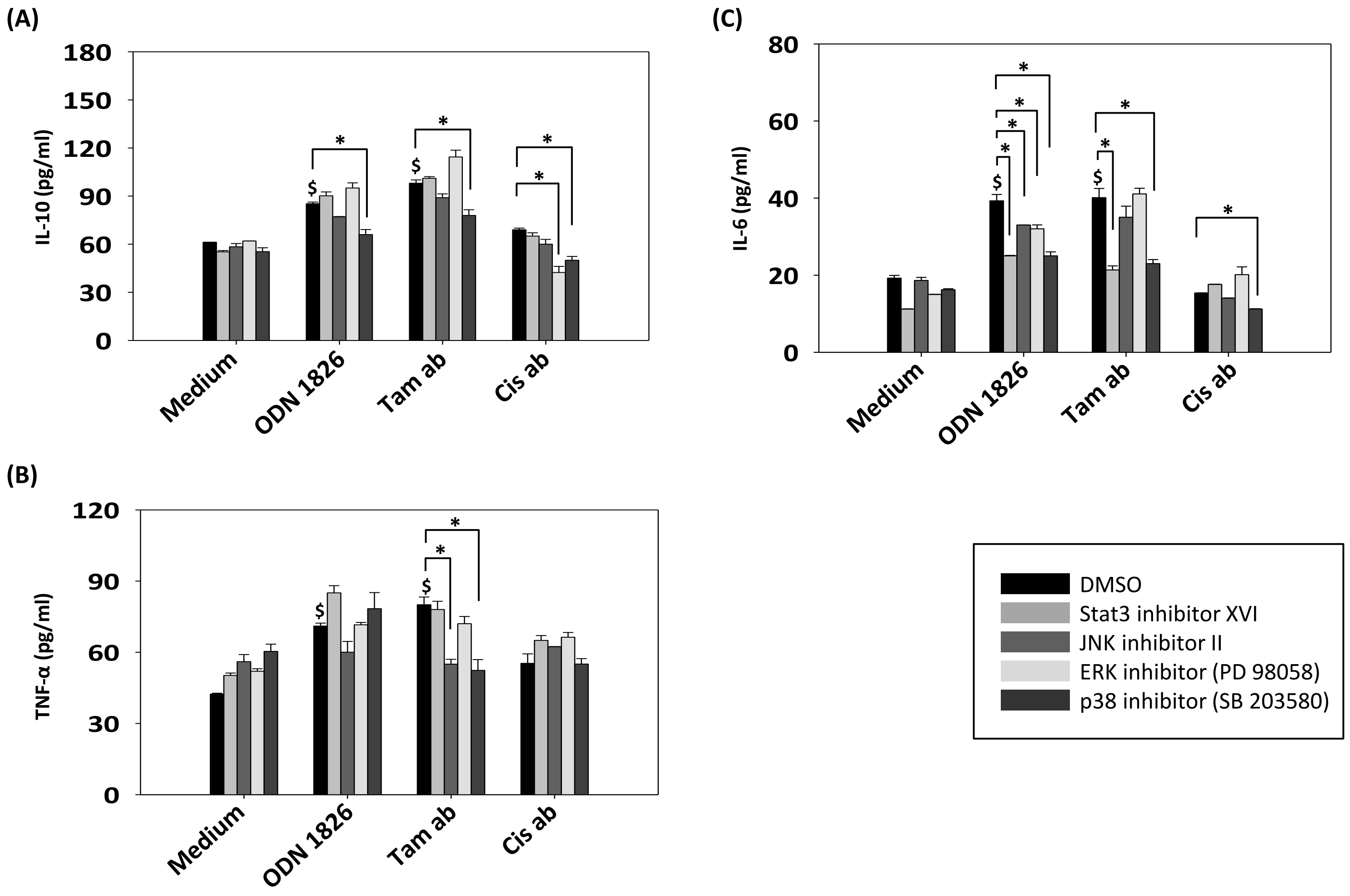

Supplementary Figure 2. Effect of signaling inhibitors on apoptotic-body induced cytokine secretion from splenocytes. Splenocytes from NZM mice were individually incubated with tamoxifen-induced apoptotic bodies (Tam ab) or cisplatin-induced apoptotic bodies (Cis ab), in the presence or absence of signaling inhibitors; DMSO was used as vehicle, and ODN 1826 was employed as positive control. (A) IL-10, (B) TNF-α and (C) IL-6 were estimated in supernatants. Bars represent arithmetic means ± SEM from three different experiments. $^{\$}p \leq 0.05$ versus Medium ;* $p \leq 0.05$ by ANOVA.

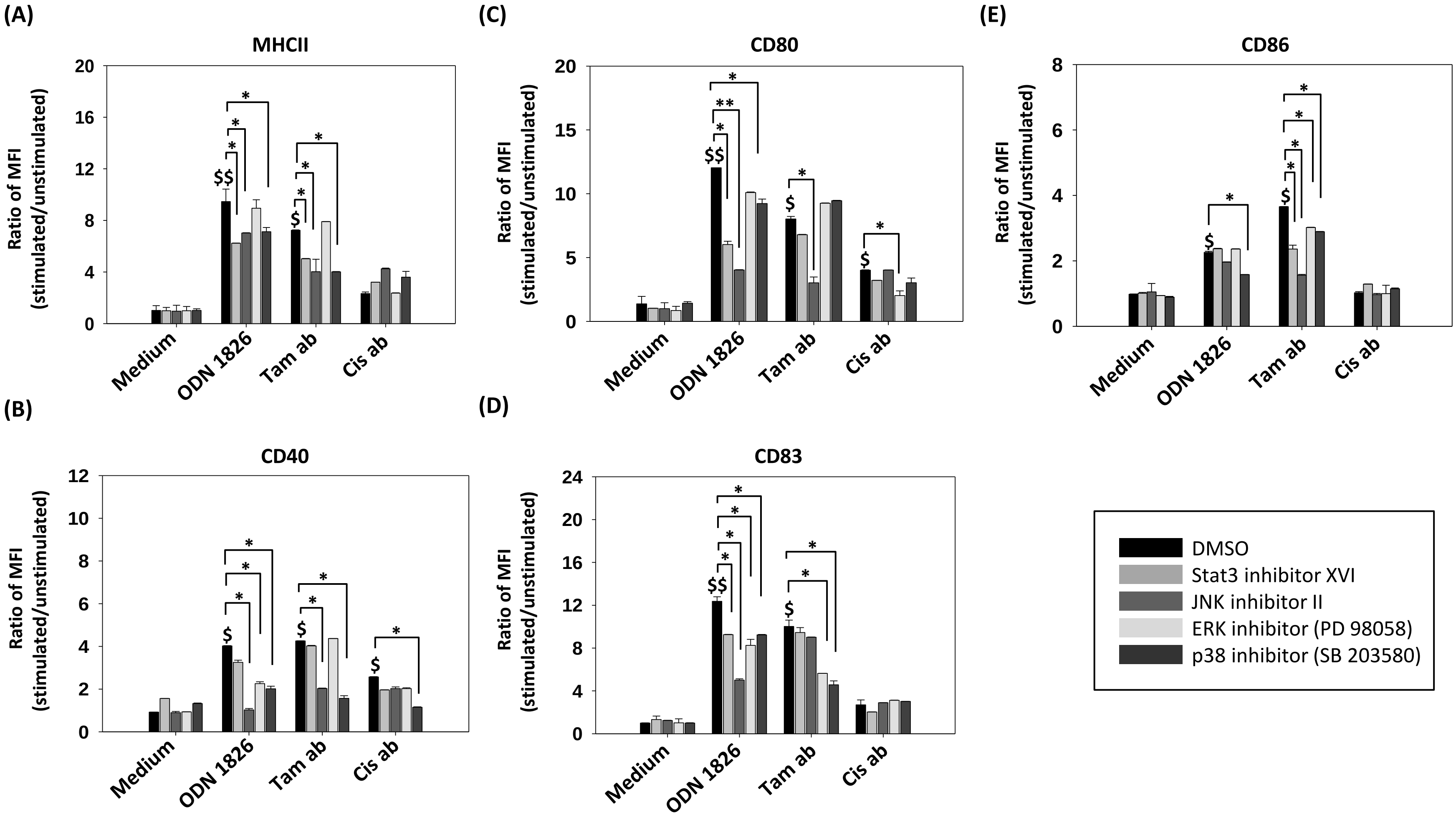

Supplementary Figure 3. Effect of signaling inhibitors on apoptotic-body induced phenotypic changes on B cells. Splenocytes from NZM mice were individually incubated with tamoxifen-induced apoptotic bodies (Tam ab) or cisplatin-induced apoptotic bodies (Cis ab), in the presence or absence of signaling inhibitors (see text for details); DMSO was used as vehicle. ODN 1826 was employed as positive control. Levels of (A) MHCII, (B) CD40, (C) CD80, (D) CD83 and (E) CD86 on $CD19^{+}$ cells was assessed by flow cytometry. Bars represent arithmetic means ± SEM of ratios (stimulated cells / unstimulated cells) of Mean Fluorescence Intensity (MFI) from three different experiments. $^{\$}p\leq0.05$, $^{\$\$}p\leq0.01$ versus Medium; $^{*}p\leq0.05$, $^{**}p\leq0.01$ by ANOVA.

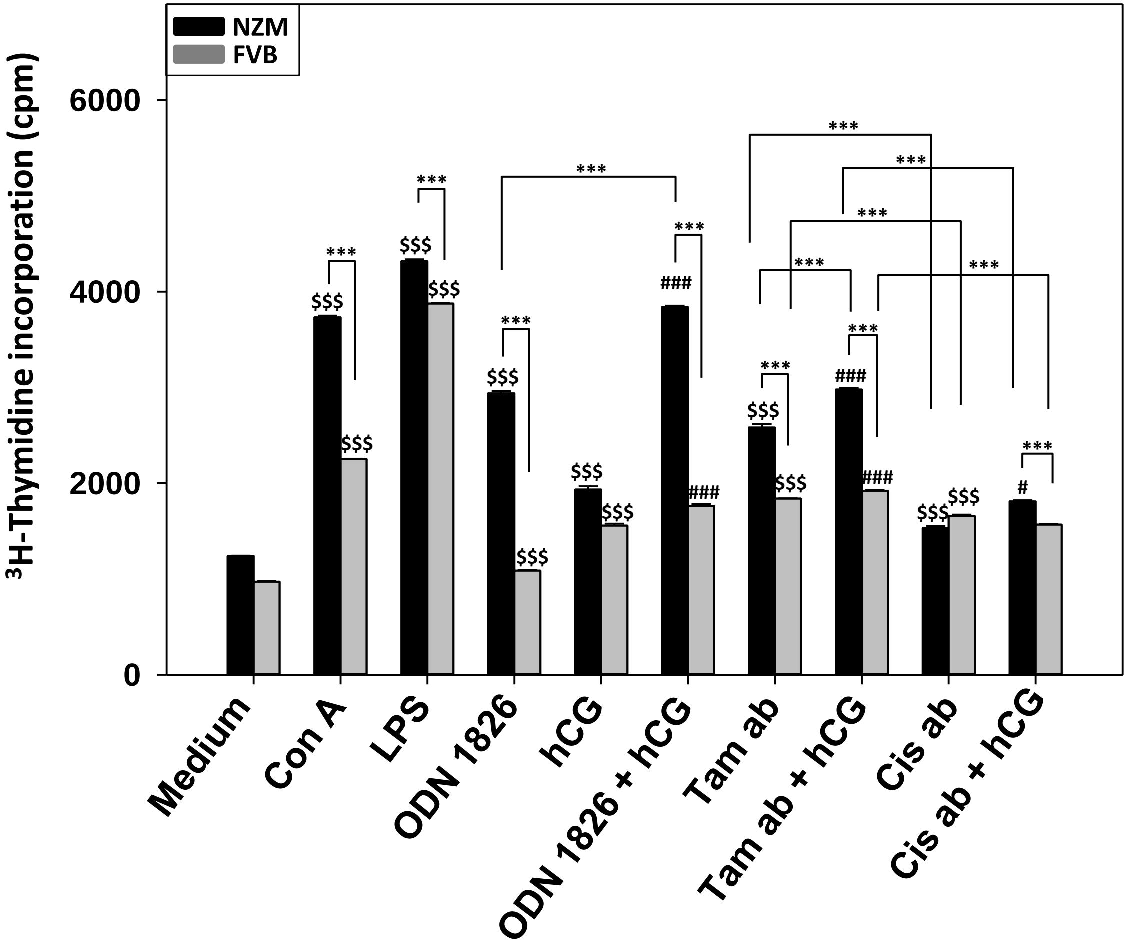

Supplementary Figure 4. Effect of hCG-apoptotic body co-incubation on proliferative responses. Splenocytes from NZM and FVB mice were incubated with hCG, tamoxifen-induced apoptotic bodies (Tam ab) , Tam ab + hCG, cisplatin-induced apoptotic bodies (Cis ab) or Cis ab + hCG. Con A, LPS and ODN 1826 were employed as positive controls. Incorporation of ^{3}H-Thymidine is depicted. Bars represent arithmetic means ± SEM of three different experiments. $^{\$\$\$}p \leq 0.001$ versus Medium; $^{\#}p \leq 0.05$, $^{\#\#\#}p \leq 0.001$ versus hCG; $^{***}p \leq 0.001$, by ANOVA. For purposes of clarity, significances versus Medium are not shown for conditions containing ODN 1826 / blebs + hCG.
